# Enhanced Skeletal Muscle Oxidative Capacity and Capillary-to-Fiber Ratio Following Moderately Increased Testosterone Exposure in Young Healthy Women

**DOI:** 10.3389/fphys.2020.585490

**Published:** 2020-12-03

**Authors:** Daniele A. Cardinale, Oscar Horwath, Jona Elings-Knutsson, Torbjörn Helge, Manne Godhe, Stéphane Bermon, Marcus Moberg, Mikael Flockhart, Filip J. Larsen, Angelica Lindén Hirschberg, Björn Ekblom

**Affiliations:** ^1^Department of Physiology, Nutrition and Biomechanics, Åstrand Laboratory, The Swedish School of Sport and Health Sciences, Stockholm, Sweden; ^2^Elite Performance Centre, Bosön - Swedish Sports Confederation, Lidingö, Sweden; ^3^Department of Women’s and Children’s Health, Karolinska Institutet, Stockholm, Sweden; ^4^Department of Gynaecology and Reproductive Medicine, Karolinska University Hospital, Stockholm, Sweden; ^5^LAMHESS, Université Côte d’Azur, Nice, France

**Keywords:** endurance performance, mitochondria, muscle morphology, oxidative, testosterone

## Abstract

**Background:** Recently, it was shown that exogenously administered testosterone enhances endurance capacity in women. In this study, our understanding on the effects of exogenous testosterone on key determinants of oxygen transport and utilization in skeletal muscle is expanded.

**Methods:** In a double-blinded, randomized, placebo-controlled trial, 48 healthy active women were randomized to 10 weeks of daily application of 10 mg of testosterone cream or placebo. Before and after the intervention, VO_2_ max, body composition, total hemoglobin (Hb) mass and blood volumes were assessed. Biopsies from the *vastus lateralis* muscle were obtained before and after the intervention to assess mitochondrial protein abundance, capillary density, capillary-to-fiber (C/F) ratio, and skeletal muscle oxidative capacity.

**Results:** Maximal oxygen consumption per muscle mass, Hb mass, blood, plasma and red blood cell volumes, capillary density, and the abundance of mitochondrial protein levels (i.e., citrate synthase, complexes I, II, III, IV-subunit 2, IV-subunit 4, and V) were unchanged by the intervention. However, the C/F ratio, specific mitochondrial respiratory flux activating complex I and linked complex I and II, uncoupled respiration and electron transport system capacity, but not leak respiration or fat respiration, were significantly increased following testosterone administration compared to placebo.

**Conclusion:** This study provides novel insights into physiological actions of increased testosterone exposure on key determinants of oxygen diffusion and utilization in skeletal muscle of women. Our findings show that higher skeletal muscle oxidative capacity coupled to higher C/F ratio could be major contributing factors that improve endurance performance following moderately increased testosterone exposure.

## Introduction

Androgens such as testosterone are essential hormones that regulate several physiological processes, including growth, cell differentiation, metabolism, the immune system, aspects of sexual and cognitive functions, and secondary sex characteristics (Herbst and Bhasin, 2004). Exogenous testosterone has been shown to improve sexual and cognitive functions (Davis and Wahlin-Jacobsen, 2015), lean mass, bone mineral density, and strength (Herbst and Bhasin, 2004). Testosterone administration is known to increase erythropoiesis *via* a direct action on bone marrow or indirectly by increasing the synthesis and secretion of erythropoietin (Shahani et al., 2009) in a dose- and concentration-dependent manner (Bhasin et al., 2001; Huang et al., 2014). Angiogenesis, endothelial function (Yoshida et al., 2015), and mitochondrial biogenesis (Usui et al., 2014) are also reported being under influence of androgens. The above-mentioned effects induced by testosterone are although reversed when testosterone administration is stopped (Forbes et al., 1992) or endogenous testosterone is suppressed (Mauras et al., 1998).

Since the physiological effects of androgens may be decisive for performance, it is not surprising that androgens have been secretly used for many years to enhance physical performance (Franke and Berendonk, 1997); androgens remain the most common class of doping agents used by athletes (IAAF, 2020). The apparent sex-based differences in athletic performance are largely attributed to the 10–20 times higher circulating levels of testosterone in men than in women (Handelsman et al., 2018; Hirschberg, 2020). Therefore, increasing the circulating levels of testosterone may potentially be more beneficial for improving physical performance in women than in men (Huang and Basaria, 2018). Nonetheless, there are few studies on the effect of supplemented testosterone in healthy women (Strauss, 1985) because it is difficult to conduct such studies, particularly in young women, for obvious ethical reasons and potential adverse effects.

In a recent double-blinded randomized placebo-controlled study, it was shown that endurance performance (i.e., time to exhaustion during a graded exercise test) in active healthy young women was enhanced by 21.17 s (8.5%), along with increased total and lower limb lean mass, by moderately increased testosterone concentrations (to a mean of 4.3 nM, which is 4.8 times higher than the baseline level) for 10 weeks compared to placebo (Hirschberg et al., 2020). This enhanced endurance performance was possibly mediated by improved muscle function, since the cross-sectional area of mixed and type II fibers was enlarged from pre to post treatment (Horwath et al., 2020). However, since neither leg muscle strength nor power were positively affected by testosterone supplementation, factors linked to oxygen transport to the muscle and skeletal muscle oxygen utilization may have contributed to enhance endurance performance.

Therefore, the present study aimed to expand on our previous findings (Hirschberg et al., 2020; Horwath et al., 2020) by examining whether oxygen consumption per muscle mass, hemoglobin (Hb) mass, blood (BV), plasma volume (PV), red blood cell volume (RCV), capillary density, capillary count per fiber and skeletal muscle mitochondrial oxidative capacity were affected by a moderate increased in circulating testosterone levels in young healthy women. It was hypothesized that a moderate increase in testosterone concentration would affect microvasculature and mitochondrial protein levels and would cause mitochondrial oxidative capacity of skeletal muscles to increase, but we did not expect it to affect oxygen consumption per lean muscle mass due to the simultaneous increase of lean muscle mass and Hb mass.

## Materials and Methods

### Ethical Approval

This study was approved by the local ethics committee in Stockholm (2016/1485-32, amendment 2017/779-32) and was conducted according to the Declaration of Helsinki. All subjects were carefully informed of the possible risks, adverse events and discomfort related to the study and signed an informed consent prior to their participation. This trial is registered at ClinicalTrials.gov (NCT03219558).

### Study Design

The present study design has previously been described elsewhere (Hirschberg et al., 2020; Horwath et al., 2020). Briefly, this was a randomized, double-blinded, placebo-controlled study performed at the Karolinska University Hospital and the Swedish School of Sport and Health Sciences between May 2017 and June 2018. All investigators, research coordinators and subjects were blinded to treatment provision. Using a balanced block-randomization, the subjects were assigned to 10 weeks of placebo or testosterone treatment. The subjects manually applied a cream every evening to the outer thigh containing either testosterone (10 mg, Andro-Feme® 1) or placebo. The testosterone dose was selected to elevate systemic testosterone levels without inducing harmful side effects (Hirschberg et al., 2020). Before and after the intervention, body composition and systemic oxygen consumption were determined, and fasting blood samples and muscle biopsies were collected.

### Subjects

Forty-eight healthy young women were recruited for the present study through local advertisements. The inclusion criteria were as follows: 18–35 years old, body mass index 19–25 kg/m^2^, non-smoking, moderate to high self-reported level of recreational physical activity, were not using hormonal contraception and were willing to use non-hormonal contraception throughout the study period. The baseline characteristics of the subjects are presented in Table 1. The presence of any medical condition, musculoskeletal injury, or an intake of hormonal contraception 2 months prior to the commencement of the study served as exclusion criteria. Baseline data were collected during the early follicular phase of the menstrual cycle (cycle days 1–7) before the start of treatment. At the end of treatment, sample collection was conducted randomly according to cycle phase. Compliance and adverse events were monitored continuously and have been documented elsewhere (Hirschberg et al., 2020). Furthermore, the subjects were instructed to maintain their physical activity levels throughout the 10-week period.

**Table 1:** Subject characteristics, hormone concentrations, lean mass, and VO_2_ max pre and post intervention.

	Testosterone (*n* = 24)		Placebo (*n* = 24)		*p* value
	Pre	Post	Pre	Post	
Age (y)	28.4 ± 3.2	28.4 ± 3.2	28.4 ± 4.3	28.4 ± 4.3	
Height (cm)	169.8 ± 5.2	169.8 ± 5.1	167.7 ± 5.7	167.9 ± 5.7	0.424
Body mass (kg)	67.1 ± 7.2	67.2 ± 7.3	65.1 ± 7.1	65.4 ± 7.1	0.875
Serum levels of testosterone (nmol L^−1^)	0.9 ± 0.4	4.3 ± 2.8^*^	1.0 ± 0.4	1.1 ± 0.4	0.001
Total lean mass (kg)	47.0 ± 4.9	47.8 ± 4.9^*^	45.4 ± 5.4	45.6 ± 5.3	0.039
Leg lean mass (kg)	15.9 ± 1.6	16.3 ± 1.6^*^	15.3 ± 1.9	15.4 ± 2.0	0.039
VO_2_ max (L min^−1^)	3.0 ± 0.4	3.1 ± 0.4^*^	2.9 ± 0.4	2.9 ± 0.4	0.29

Values are the means ± SD. The measurements were performed pre and post 10 weeks of either 10 mg of testosterone cream daily or placebo. The between-groups level of significance is numerically presented.

* = *p* < 0.05 for within-group analyses.

### Hormone Concentrations

Blood samples pre and post intervention were collected in a rested state after an overnight fast. After centrifugation, the serum was separated and stored at −80°C until analysis. Serum testosterone levels were assessed with gold-standard liquid chromatography-tandem mass spectrometry (LC-MS/MS). Follicle stimulating hormone, luteinizing hormone, and sex hormone binding globulin were determined by electrochemiluminescence immunoassay (Eklund et al., 2017). Free androgen index was calculated [testosterone (nmol/L)] divided by sex hormone binding globulin [(nmol/L) x 100] as described earlier (Hirschberg et al., 2020).

### Maximal Oxygen Consumption

The maximal oxygen consumption (VO_2_ max) test was performed as described previously (Hirschberg et al., 2020). Briefly, the running test was performed to exhaustion on a treadmill (Rodby Electronics, Hagby Sweden) starting at either 10 or 12 km/h with a 1° incline (determined from the results of a previous familiarization test). The treadmill speed increased by 1 km/h every min for the first 4 min, after which the workload was increased by raising the incline 1° every following min until exhaustion. Time to exhaustion was registered as the time in s from the start of the running test protocol until the subject voluntarily stopped or failed to safely keep up with the treadmill speed. Respiratory gases in the expired air were measured continuously using an on-line ergo spirometry with a mixing chamber system (Oxycon Pro, Erich Jaeger GmbH, Hoechberg, Germany). System calibration was performed according to the manufacturer’s instructions before each test. The criteria for reaching VO_2_ max were as follows: no increase in VO_2_ at increased workload (leveling off), work time higher than 4 min, respiratory exchange ratio higher than 1.05 and rating perceived exertion higher than 17 on a scale 6–20 (Borg, 1970). The peak VO_2_ and heart rate values were expressed as the highest mean values over a 30-s period from the measurement recordings.

### Body Composition

As described elsewhere (Hirschberg et al., 2020), lean mass (total and legs) and fat mass were determined at both pre and post intervention with dual energy X-ray absorptiometry (Lunar Prodigy Advance, GE Healthcare, United States).

### Total Hemoglobin Mass and Blood Compartment Volumes

The total Hb mass and blood compartment volumes were assessed as described elsewhere (Burge and Skinner, 1995) with some minor modifications of the re-breathing technique. Briefly, with the participants still lying on the bench in a semi-recumbent position following the biopsy collection, 15 ml of blood was sampled from an antecubital vein *via* a 20-gage Venflon and was immediately analyzed to assess the Hb concentration using the HemoCue® Hb 201+ System (HemoCue AB, Ängelholm, Sweden) and the Hematocrit (Hct) concentration in quadruplet *via* the micro-method (3 min at 13,500 rpm). One and a half milliliters of the blood sample were then quickly transferred to a 2-ml Eppendorf tube and stored at −80°C until the percent carboxyhemoglobin (%HbCO) was analyzed using an haemoximeter (ABL800, Radiometer, Copenhagen, Denmark). After baseline collection, the participants breathed from a Douglas bag previously filled with pure oxygen for 4 min to remove nitrogen from the airways. During this time, the operator flushed the re-breathing circuit with pure oxygen, which was then closed. After 4 min, the operator switched the participant to the re-breathing circuit, and a precisely measured bolus of 0.8 ml·kg^−1^ body mass of 99.997% chemically pure CO (CO N47, Air Liquide, Paris, France) was injected into the circuit. The participants then breathed the gas mixture for 10 min. Thereafter, an additional venous blood sample was collected from an antecubital vein to assess the change in %HbCO accounting for the CO remaining in the re-breathing circuit, which was determined using the Monoxor III (Bacharach Inc., New Kensington, United States). The Hb mass was calculated from the change in %HbCO, and the total RCV, BV, and PV were derived (Burge and Skinner, 1995).

### Muscle Biopsy Sampling

Pre and post muscle samples were collected (~60 min) after the participants had finished a battery of performance tests, as described previously (Horwath et al., 2020). Muscle biopsies were obtained from the middle portion of the vastus lateralis muscle after the administration of local anesthesia (2–4 ml of 20 mg ml^−1^ Carbocaine; Astra Zeneca, Södertälje, Sweden). Using the Weil-Blakesley conchotome technique (Ekblom, 2017), one muscle sample (~50 mg) was snap frozen in liquid nitrogen for later analysis of the mitochondrial proteins. Another sample was quickly dissected free from visible blood and connective tissue, embedded in OCT-medium and frozen in isopentane cooled by liquid nitrogen. This specimen was later used for the immunohistochemical analyses. A third sample (~10 mg) was immediately placed in ice-cold mitochondrial isolation medium upon removal (100 mM sucrose, 100 mM KCl, 50 mM Tris-HCl, 1 mM KH_2_PO_4_, 100 μM EGTA, 0.1% BSA; final pH of 7.4), followed by the mitochondria isolation procedure (described later in the methods section).

### Protein Extraction and Immunoblot Analysis

To analyze the levels of specific mitochondrial proteins in the muscle, 2 mg of lyophilised muscle was homogenized in ice-cold buffer (100 μlmg^−1^ dry weight) consisting of 2 mM HEPES (pH 7.4), 1 mM EDTA, 5 mM EGTA, 10 mM MgCl_2_, 50 mM β-glycerophosphate, 1% TritonX-100, 2 mM dithiothreitol, 1% phosphatase inhibitor cocktail (Sigma P-2850) and 1% (v/v) Halt Protease Inhibitor Cocktail (Thermo Scientific, Rockford, United States) using a Bullet Blender™ (NextAdvance, New York, United States). The resulting homogenates were rotated for 60 min at 4°C and subsequently centrifuged at 10,000 *g* for 10 min at 4°C. The supernatant was collected, and the protein concentrations were determined in an aliquot of the supernatant using the Pierce™ 660 nm protein assay (Thermo Scientific, Rockford, United States). The samples were diluted in 4x Laemmli sample buffer (Bio-Rad Laboratories, Richmond, CA, United States) and homogenizing buffer to obtain a final protein concentration of 0.75 μg μl^−1^. The samples were then heated at 95°C for 5 min to denature the proteins. The samples were stored at −20°C until separation on SDS-PAGE.

For protein separation, 15 μg of protein from each sample was loaded onto 26-well Criterion TGX gradient gels (4–20% acrylamide; Bio-Rad Laboratories), with all samples from each subject loaded onto the same gel, and electrophoresis was performed on ice at 300 V for 30 min. Next, the gels were equilibrated in transfer buffer (25 mM Tris base, 192 mM glycine, and 10% methanol) for 30 min at 4°C, after which the proteins were transferred to polyvinylidene fluoride membranes (Bio-Rad Laboratories) at a constant current of 300 mA for 3 h at 4°C. Equal loading and transfer were then confirmed by staining the membranes with MemCode™ Reversible Protein Stain Kit (Thermo Scientific).

Following destaining, the membranes were blocked for 1 h at room temperature in Tris-buffered saline (TBS; 20 mM Tris base, 137 mM NaCl, pH 7.6) containing 5% nonfat dry milk and then incubated overnight with commercially available primary antibodies diluted in TBS supplemented with 0.1% Tween-20 containing 2.5% non-fat dry milk (TBS-TM). The following morning, the membranes were washed with TBS-TM and incubated for 1 h at room temperature with secondary antibodies conjugated with horseradish peroxidase. Next, the membranes were washed with TBS-TM (2 × 1 min, 3 × 10 min), followed by 4 × 5 min with TBS. Finally, the proteins were visualized by applying Super Signal™ West Femto Chemiluminescent Substrate (Thermo Scientific) to the membranes, followed by detection in the molecular imager ChemiDoc™ MP and quantification of the detected bands using the Image Lab™ software (Bio-Rad Laboratories). Prior to blocking, the membranes from each gel were cut into stripes for each target protein and then assembled. Thus, all membranes with samples from one subject were exposed to the same blotting conditions. The proteins were normalized to the corresponding total protein stain (entire lane) for each sample. Primary antibodies against citrate synthase (#96600; 1:2,000) and OXPHOS (#110413; 1:2,000) were purchased from Abcam (Cambridge, United Kingdom), and primary antibodies against COX IV (#4850; 1:2,000) were purchased from Cell Signaling Technology (Beverly, MA, United States). Secondary anti-rabbit (#7074; 1:10,000) and secondary anti-mouse (#7076; 1:10,000) antibodies were purchased from Cell Signaling Technology.

### Immunohistochemistry

**Figure 1:**
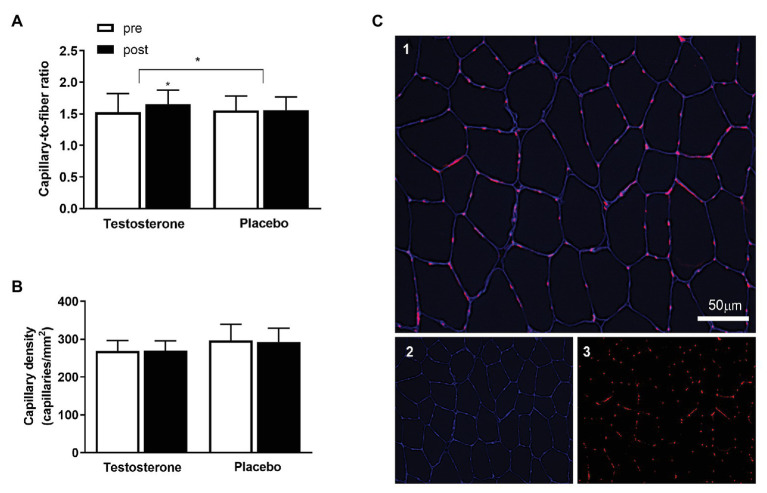
The **(A)** displays the capillary-to-fiber (C/F) ratio and **(B)** the capillary density pre (white bars) to post intervention (black bars) in the testosterone (*n* = 18) and placebo (*n* = 15) groups. Data are presented as means ± SD. The **(C)** displays the immunohistochemical labeling of capillaries in the muscle cross-section. Merged image of CD31 in red and laminin in blue **(C1)**, single channel laminin in blue **(C2)**, and single channel CD31 in red **(C3)**. The white scale bar = 50 μm. ^*^ = *p* < 0.05. The between-group differences in the changes from pre to post intervention with the baseline measurement as a covariate are indicated with the bracket and the ^*^ symbol. The ^*^ symbol over the bar indicates a within-group difference from pre to post intervention.

Seven-micrometer-thick cryosections were cut from the OCT-embedded biopsy specimen using a cryostat (Leica CM1950). The cryosections were then mounted onto microscope glass slides (VWR International), air-dried for 1 h, and stored at −80°C. Pre and post samples were placed on the same glass slide to minimize variations in staining efficiency. To stain the muscle capillaries, the slides were fixed in 4% paraformaldehyde (PFA), washed in phosphate buffered saline (PBS) and thereafter incubated for 2 h with primary antibodies against laminin (1:50; D18, DSHB, United States) and CD31/PECAM (1:400; JC70, Santa Cruz Biotechnology, United States) diluted in PBS containing 5% normal goat serum (NGS) and 0.02% Triton X-100. After being washed in PBS, secondary antibodies (1:500; 350 goat anti-mouse IgG2A and 1:1,000; 594 goat anti-mouse IgG1, Alexa Fluor, Invitrogen, United States) diluted in PBS containing 1% NGS were applied. The slides were mounted with a cover slip and Prolong Gold Antifade Reagent (Invitrogen, United States). This protocol stained the cell borders in blue and the muscle capillaries in red, as depicted in Figure 1.

### Image Acquisition and Analysis

The images were captured using a fluorescent widefield microscope (Celena® S, Logos Biosystems, South Korea) with a ×10 objective. The analyses were performed using ImageJ software (National Institutes of Health, United States), and the quantification of muscle capillarization was performed in accordance with previous studies (Charifi et al., 2004). Capillary density was determined by calculating the number of capillaries in a given area, expressed as capillaries per mm^2^. Furthermore, the capillary-to-fiber ratio (C/F) was assessed by calculating the number of capillaries and the number of fibers present within the same area, applying the recommended correction (Brodal et al., 1977). Analyses of the capillary network contained an average of 206 ± 60 fibers (SD) per biopsy and included 33 participants (placebo; *n* = 15, testosterone; *n* = 18). All immunofluorescence analyses were performed by a single investigator in a blinded manner.

### Isolation of Mitochondria

Mitochondrial isolation was conducted as previously published (Cardinale et al., 2018b). Briefly, the muscle biopsy was first weighed and then cut in ice-cold isolation medium (100 mM sucrose, 100 mM KCl, 50 mM Tris-HCl, 1 mM KH_2_PO_4_, 100 μM EGTA, 0.1% BSA; final pH of 7.4; Gnaiger and Kuznetsov, 2002). One milliliter of isolation medium containing 0.2 mg ml^−1^ bacterial protease was then added to the homogenate and transferred to a glass jacket connected to an ice-cold bath pump for further homogenization with a handheld electric drill (80 rpm). The final homogenate was centrifuged at 700 *g* at 4°C for 10 min. The supernatant was again centrifuged at 10,000 *g* at 4°C, and the resultant mitochondrial pellet was re-suspended in the same medium. The Eppendorf tube was then centrifuged at 7,000 *g* for 5 min, and the pellet was dissolved in 0.6 μl of preservation medium (0.5 mM EGTA, 3 mM MgCl_2_6H_2_O, 60 mM K-lactobionate, 20 mM Taurine, 10 mM KH_2_PO_4_, 20 mM HEPES, 110 mM sucrose, 1 gl^−1^ BSA, 20 mM histidine, 20 μM vitamin E succinate, 3 mM glutathione, 1 μM leupeptin, 2 mM glutamate, 2 mM malate, and 2 mM Mg-ATP; Gnaiger and Kuznetsov, 2002) per mg of original tissue.

Mitochondrial respiration was performed in a two-channel high-resolution respirometer (Oroboros Oxygraph, Paar, Graz, Austria). Data sampling was set for 1 s intervals and averaged over 40 s. Each experiment was run twice, and the O_2_ flux values of the two chambers were then averaged. MiR05 medium (0.5 mM EGTA, 3 mM MgCl_2_.6H_2_O, 60 mM K-lactobionate, 20 mM taurine, 10 mM KH_2_P0_4_, 20 mM HEPES, 110 mM sucrose, and 1 g·l^−1^ BSA) was used to assess respiration. All experiments were performed at 37°C. O_2_ consumption and zero-drift of the O_2_ electrode were calculated using DatLab 5.2 software (Oroboros, Paar, Graaz, Austria).

### Mitochondrial Respiratory Protocols

Mitochondrial respiration was measured by titrating the substrates into the chambers to assess leak respiration (L) with malate (2 mM), palmitoylcarnitine (0.2 mM), and pyruvate (5 mM) in the absence of adenylates; complex I respiration (CI_P_) was assessed with the addition of saturating ADP (5 mM); and convergent complex I + II linked ADP-stimulated maximal respiration (CI + II_P_) was assessed with the addition of succinate (10 mM). Carbonyl cyanide m-chloro phenyl hydrazine (0.05 μM steps) was used to measure maximal uncoupled oxidative phosphorylation (Unc). N,N,N',N'-Tetramethyl-p-phenylenediamine dihydrochloride (0.5 mM) and ascorbate (2 mM) followed by sodium azide (100 mM) were used to assess cytochrome C oxidase activity. Mitochondrial respiration rates (pmols^−1^) were normalized for mitochondrial suspension protein levels (pmols^−1^ μg^−1^) in the isolated mitochondria preparation using the Pierce 660 nm protein assay (Thermo Scientific) to obtain the intrinsic mitochondrial respiration, which is an index of mitochondrial quality.

### Statistics

The results are presented as the mean ± SD. The data were initially tested for normality and equal variance using the Shapiro–Wilk test of normality and Q-Q plots. An analysis of covariance (ANCOVA) adjusting for baseline variation and the Bonferroni *post hoc* procedure for multiple comparisons was used to assess the between-group differences and *p* value is reported in the text. One-way ANOVA was also conducted on the same dataset using the variable changes pre to post intervention. The use of one-way ANOVA or ANCOVA did not influence the interpretation of the study results. A paired sample Student’s *t*-test was used to assess within-group differences from pre to post treatment. Correlations between variables were calculated using Spearman’s rank correlation. A two-tailed value of *p* < 0.05 was considered significant. The statistical analyses were performed using SPSS statistical software version 26 (SPSS Inc., Chicago, IL, United States). The statistical analyses were blinded to the experimental conditions. The data that support the findings of this study are available from the corresponding author upon reasonable request.

## Results

### Maximal Oxygen Consumption Overall and According to Leg Lean Mass

**Figure 2:**
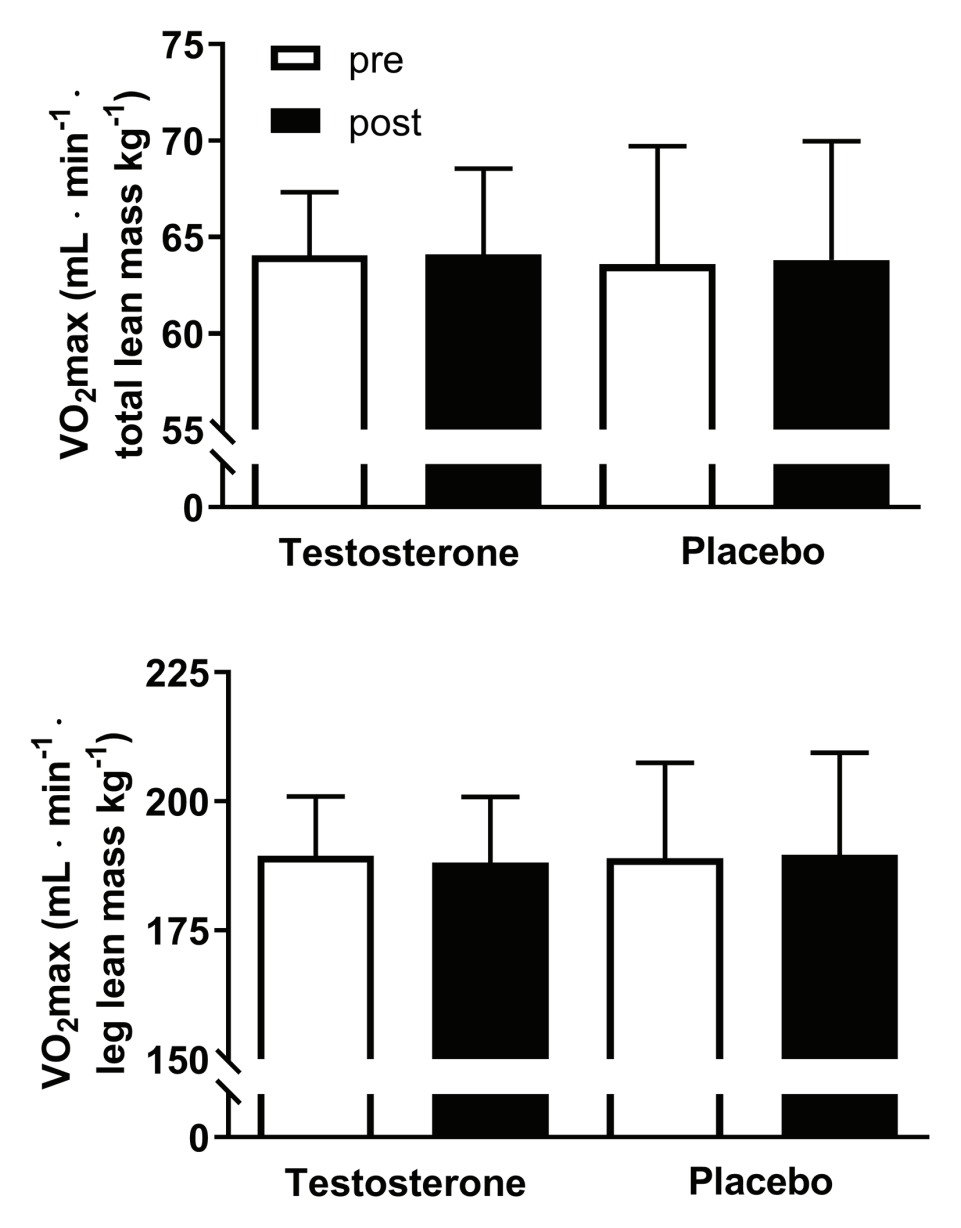
Maximal oxygen consumption (VO_2_ max) adjusted for total and leg lean mass pre (white bars) and post (black bars) 10 weeks of either 10 mg of testosterone cream daily (*n* = 24) or placebo (*n* = 24). VO_2_ max is presented as the mean ± SD and in ml min^−1^ lean mass kg^−1^.

There were no significant changes within or between groups in VO_2_ max overall and according to leg lean mass (Figure 2). However, it is important to note that the maintained VO_2_ max per kilogram muscle mass in the testosterone group was achieved by a parallel significant increase in lean mass (from 47.0 ± 4.9 kg to 47.8 ± 4.9 kg) and VO_2_ max (from 3.0 ± 0.4 L to 3.1 ± 0.4 L min^−1^) whereas these variables remained unchanged in the placebo group (Table 1), as previously reported (Hirschberg et al., 2020).

### Hematology

The hematology variables are presented in Table 2. Hb mass along with BV, PV, and RCV were unaltered by the intervention, and no differences were detected between groups. Hct levels increased significantly in the testosterone group from pre to post intervention, but no differences were detected between groups.

**Table 2:** Hemoglobin (Hb) mass and intravascular volumes before and after the intervention.

	Testosterone (*n* = 15)		Placebo (*n* = 13)		*p* value
	Pre	Post	Pre	Post	
Hb (g L^−1^)	12.4 ± 1.1	12.7 ± 1.0	13.0 ± 0.6	13.0 ± 1.0	0.62
Hct (%)	39.7 ± 2.4	40.8 ± 2.1^*^	41.1 ± 1.5	41.4 ± 1.2	0.90
Hb mass (g)	661.7 ± 108.8	693.2 ± 93.3	657.2 ± 114.5	659.5 ± 112.5	0.40
RCV (ml)	2112.1 ± 618.8	2220.6 ± 798.7	2071.5 ± 346.9	2094.2 ± 342.5	0.32
PV (ml)	3201.6 ± 375.4	3246.4 ± 535.8	2972.8 ± 522.4	2978.5 ± 567.0	0.30
BV (ml)	5313.7 ± 618.8	5467.0 ± 798.7	5044.3 ± 857.1	5072.7 ± 888.0	0.37

Values are the means ± SD. Hb, hemoglobin concentration; Hct, hematocrit; Hb mass, total hemoglobin mass; RCV, total red blood cell volume; PV, plasma volume; BV, blood volume. The measurements were performed pre and post 10 weeks of either 10 mg of testosterone cream daily or placebo. The between-groups level of significance is numerically presented.

* = *p* < 0.05 for within-group analyses.

### Microvasculature

The C/F ratio was significantly (*p* = 0.049) increased by 8% following testosterone administration compared with placebo (Figure 1A). However, the number of capillaries in a given area (i.e., capillary density; Figure 1B) was unaltered by testosterone because of the parallel increase in capillary number and cross-sectional area (Horwath et al., 2020).

### Mitochondrial Protein Abundance in Skeletal Muscle

**Figure 3:**
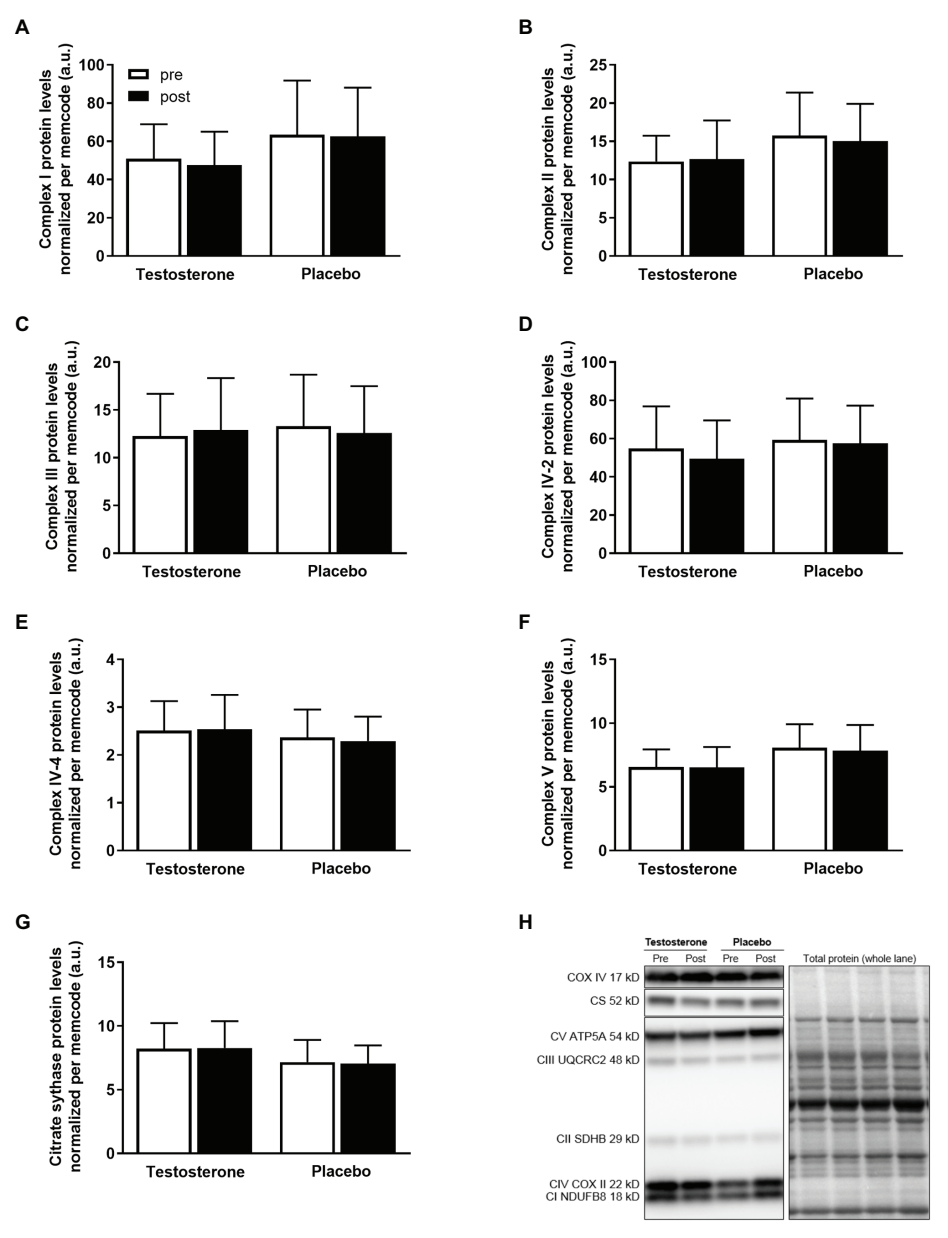
Protein levels of complex I **(A)**, complex II **(B)**, complex III **(C)**, complex IV-subunit 2 **(D)**, complex IV-subunit 4 **(E)**, complex V **(F)**, and representative blots **(G)** pre (white bars) to post intervention (black bars) in the testosterone (*n* = 18) and placebo (*n* = 17) groups. Data are presented as means ± SD. The target proteins **(H)** were expressed relative to the total lane protein stained with reversible protein stain (memcode). No between- or within-group differences were detected.

There were no significant changes in the citrate synthase, complex I, complex II, complex III, complex IV-subunit 2 and 4, or complex V protein levels in either of the treatment groups (Figure 3).

### Mitochondrial Oxidative Capacity of Skeletal Muscle

**Figure 4:**
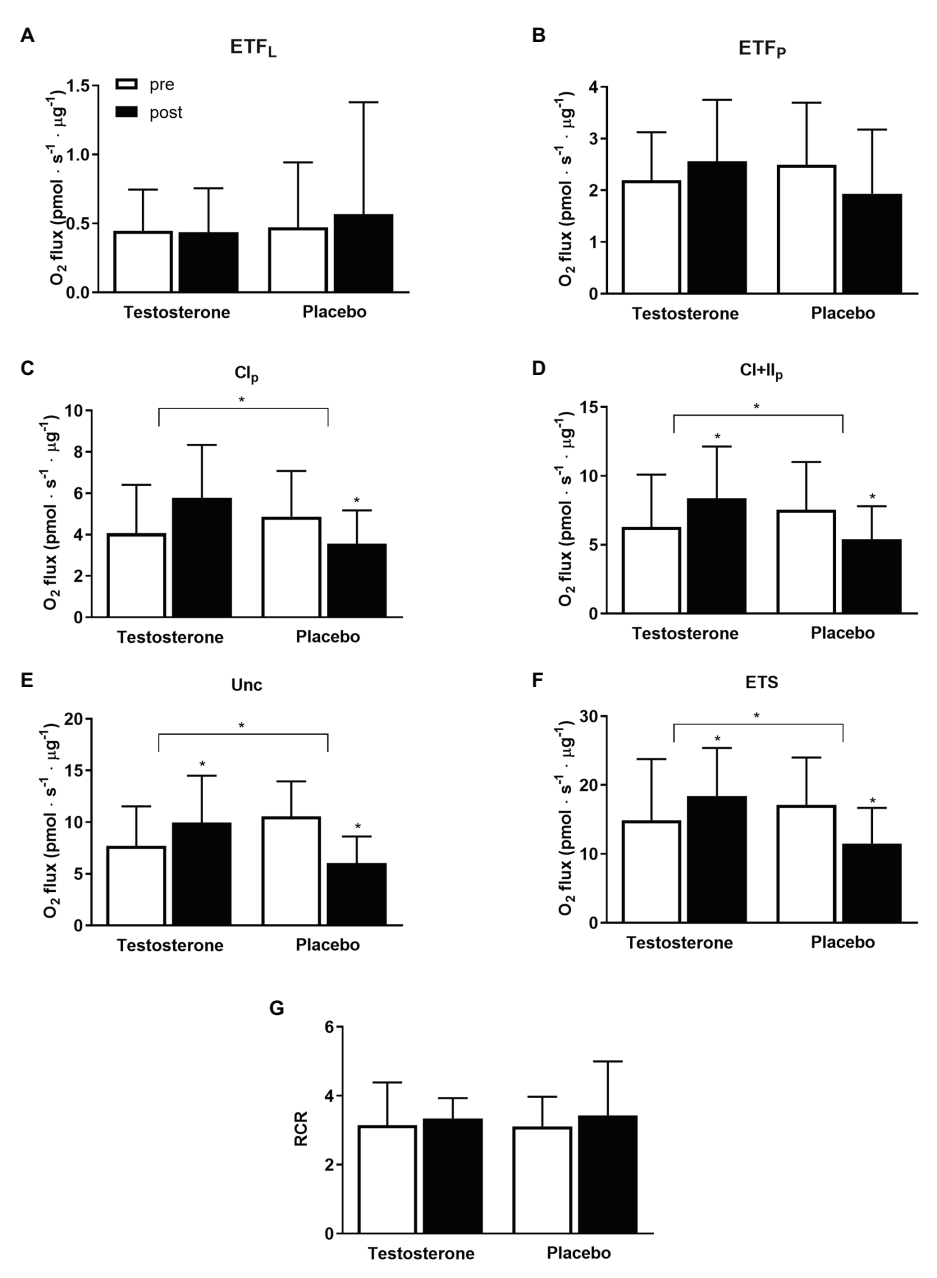
Specific mitochondrial respiratory flux from the measurement of isolated mitochondria preparation pre (white bars) and post 10 weeks (black bars) of either 10 mg of testosterone cream daily or placebo. O_2_ flux is presented as means ± SD in pmols^−1^ μg^−1^ protein. Leak respiration (**A** – ETF_L_; *p* = 0.62, testosterone *n* = 17, placebo *n* = 15); mitochondrial respiratory flux activating electron-transferring flavoprotein complex (**B** – ETF_P_; *p* = 0.12, testosterone *n* = 17, placebo *n* = 15); complex I (**C** – CI; *p* = 0.005, testosterone *n* = 17, placebo *n* = 15); linked complex I and II (**D** – CI + II; *p* = 0.004, testosterone *n* = 19, placebo *n* = 15); uncoupling state (**E** – Unc; *p* = 0.004; testosterone *n* = 14, placebo *n* = 10); electron transport system capacity (**F** – ETS; *p* = 0.001, testosterone *n* = 18, placebo n = 10), and respiratory control ratio **(G)** are reported. ^*^ = *p* < 0.05. Between-group differences in the changes from pre to post treatment with the baseline measurement as a covariate are indicated with the bracket and the ^*^ symbol. The ^*^ symbol over the bar indicates a within-group difference in the pre to post measurements.

Specific mitochondrial respiration with substrates activating complex I (*p* = 0.005), linked complex I and II (*p* = 0.005), uncoupled respiration (*p* < 0.004), and electron transport system capacity (*p* = 0.0001) were significantly higher following testosterone administration compared with placebo (Figure 4). Within-group differences were detected with the testosterone group displaying a significantly increased mitochondrial respiratory flux from pre to post intervention that ranged between 17 and 42%; conversely, the placebo group showed decreases in these variables of the same magnitude.

### Correlations

When the data from both groups where pooled together, the change in specific mitochondrial respiration activating complex I and II correlated positively with changes in serum testosterone concentrations (*r* = 0.46, *p* = 0.006), and free androgen index (*r* = 0.43, *p* = 0.01). The change in specific mitochondrial respiration activating complex I and II correlated negatively with changes in serum concentration of follicle-stimulating hormone (*r* = −0.64, *p* < 0.001) and luteinizing hormone (*r* = −0.45, *p* = 0.008). Changes in endurance performance (i.e., time to exhaustion during a graded exercise test) were positively correlated with changes in capillary density (*r* = 0.42, *p* = 0.01), and changes in complex IV-subunit 2 (*r* = 0.40, *p* = 0.02) and complex V (*r* = 0.36, *p* = 0.04) protein levels.

## Discussion

This study provides novel insights regarding the physiological actions of increasing the level of circulating testosterone on key determinants of oxygen transport and utilization in the skeletal muscle of physically active healthy young women.

It has been previously shown that 10 mg of testosterone supplemented daily for 10 weeks elevated the circulating levels of testosterone to a mean level of 4.3 nM (4.8 times higher than the baseline level) causing endurance performance to improve by 8.5%, along with an increased total and lower limb lean mass compared with placebo (Hirschberg et al., 2020). Muscle strength and power output measures were not affected by the intervention even though muscle fiber hypertrophy was observed in women undergoing testosterone treatment particularly evident in the type II fibers, which displayed a 9.2% increase in fiber cross-sectional area after 10 weeks (from 4,952 ± 1,168 to 5,407 ± 1,189 μm^2^), as reported elsewhere (Horwath et al., 2020).

The present investigation demonstrated that compared to placebo, the C/F ratio in skeletal muscle was augmented following testosterone supplementation. A higher C/F ratio not only increases the capillary bed surface available for oxygen and metabolite diffusion but also increases capillary mean transit time of erythrocytes, thus facilitating oxygen extraction. Therefore, it is plausible that oxygen diffusion from the microvasculature to myocytes was improved following the intervention. An increased diffusion capacity has been argued to be an important factor limiting whole body VO_2_ max (Cano et al., 2013). Despite the 8.2% increase in C/F ratio with testosterone supplementation, VO_2_ max relative to lean mass was unchanged, providing evidence against the hypothesis that diffusion capacity is a major limiting factor in the oxygen cascade at maximal work rates.

Moreover, this study showed that the skeletal muscle oxidative capacity was enhanced by testosterone administration. The changes in specific mitochondrial respiration activating complex I and II pre to post intervention were positively correlated with the changes in serum testosterone concentrations and free androgen index. Skeletal muscle oxidative capacity coupled to ATP resynthesis mainly depends on the mitochondrial content, mitochondrial quality and the ATP/O_2_ ratio if the substrate and oxygen availability are assumed not to be limiting factors. The abundance of several proteins of the electron transport system, which relates to mitochondrial content (Larsen et al., 2012), were unchanged from pre to post intervention. Conversely, the values of mitochondrial respiration activating complex I, linked complex I and II, uncoupled respiration and electron transport system capacity normalized per mitochondrial protein (i.e., mitochondria specific respiration) were all significantly higher following testosterone supplementation compared to placebo. These results indicate that the skeletal muscle oxidative capacity was enhanced by an increase in intrinsic mitochondrial respiration more than an upregulation of mitochondrial content. Although mitochondrial oxidative capacity is usually reported increased following endurance training by an increased mitochondrial content more than a higher intrinsic mitochondrial respiration (Bartlett et al., 2017; Macinnis et al., 2017), a dissociation between these two parameters has been previously reported in humans (Granata et al., 2016; Larsen et al., 2016, 2018; Lundby et al., 2018). It can be speculated that the testosterone treatment may have altered the protein aggregations of the mitochondrial respiratory complexes i.e., supercomplexes (Schagger and Pfeiffer, 2000) and/or increased mitochondrial cristae density (Nielsen et al., 2017) thus enhancing intrinsic mitochondrial respiration as it has been observed following exercise training (Greggio et al., 2017).

The abundance of mitochondrial protein in skeletal muscle was not significantly affected by testosterone administration compared with the placebo. However, the changes in endurance performance pre to post intervention were positively correlated with changes in complex IV-subunit 2 and complex V protein level. Animal studies have indicated that testosterone augments the expression of the proliferator-activated receptor-c coactivator-1α, which is a master regulator of mitochondrial biogenesis, (Wang et al., 2015) thereby increasing the protein levels of cytochrome c oxidase, specifically in muscle tissue (Usui et al., 2014). However, studies on the effects of testosterone replacement therapy in elderly men with subnormal circulating testosterone levels did not identify improved oxidative markers of mitochondrial oxidative phosphorylation (Petersson et al., 2014) and mitochondrial content (Jensen et al., 2018). Previous studies have shown that the physiological actions of exogenous testosterone are dose- and concentration-dependent in men and postmenopausal women (Bhasin et al., 2001; Huang et al., 2014). Therefore, the 10-mg daily dose of testosterone in the current group of active healthy young women may have been too low to induce significant changes in mitochondrial content. Healthy women may also respond differently to moderate elevations of circulating testosterone compared to men since physiological sexual-dimorphism in human skeletal muscle mitochondria has been previously reported (Cardinale et al., 2018b).

The increased mitochondrial oxidative capacity of the skeletal muscle in this study induced by testosterone supplementation may appear of low importance since the mitochondrial oxidative capacity has been shown to be in excess of the maximal capacity for oxygen delivery of the cardiorespiratory system (Boushel and Saltin, 2013). Instead, the excess oxidative capacity of skeletal muscle mitochondria has been shown to directly affect oxygen extraction between microvasculature and myocytes (Cardinale et al., 2019) and thus is important for endurance performance (Jacobs et al., 2011; Skattebo et al., 2020). However, as previously reported in humans (Granata et al., 2016) the changes in mitochondrial oxidative capacity in this cohort were not correlated to changes in endurance performance.

Contrary to what it was expected, the women in the placebo group manifested a reduction in mitochondrial oxidative capacity from pre to post intervention, which was consistent in most of the measured mitochondrial respiratory flux states. The reduction in mitochondrial respiration was larger than the typical error of the measurement reported in the literature (Cardinale et al., 2018a; Sahl et al., 2018). Since the subjects were block-randomized and technicians were blinded to the treatment, it is highly unlikely that mitochondrial sample preparations, contamination of samples, substrates fed to the mitochondria or the performance of high-resolution respirometers influenced these results. Although unlikely, the testosterone supplementation might have influenced behavioral changes such as physical activity levels whereas the placebo group spontaneously reduced their physical activity levels despite being instructed to maintain normal physical activity. However, this speculation cannot be substantiated by accelerometer data collected 1 week before the intervention and at week 10 (data not shown). We also cannot exclude the potential effect of variations in the menstrual cycle phase at the end of treatment, despite this effect on muscle metabolism being shown to be small (Devries et al., 2006).

In this study, it was also showed that VO_2_ max relative to both total and leg lean mass was preserved from pre to post testosterone intervention despite a significant increase in the total and leg lean mass following testosterone treatment (Hirschberg et al., 2020). Parameters such as BV, PV, and RCV, which are known to increase venous return and preload to the heart and thus can affect cardiac output, (Hopper et al., 1988; Bonne et al., 2014) were unaltered from pre to post intervention. Testosterone regulates erythropoiesis and may have directly and indirectly induced increases in Hb mass (Shahani et al., 2009). However, the preserved VO_2_ max per lean muscle mass following testosterone treatment was not achieved by an increased oxygen carrying capacity of blood since no significant change was detected in the total Hb mass from pre to post intervention. Instead, it is plausible that the combined increase of skeletal muscle oxidative capacity and C/F ratio led to improved oxygen extraction compared to placebo, as discussed above.

In conclusion, it is here demonstrated for the first time that increasing the circulating testosterone to levels below 5 nmol/L, which is the threshold set in World Athletics eligibility regulations for the female classification, improves the microvasculature and the oxidative capacity of skeletal muscle, thus preserving VO_2_ max despite a significant increase in lean muscle mass in physically active healthy young women. This study provides novel insight regarding the physiological effects of increased circulating testosterone levels on endurance performance in active healthy young women. These results support the body of evidence on the effects of testosterone in women (Hirschberg et al., 2020; Horwath et al., 2020) and help to put light on the controversy regarding eligibility regulations in athletes with naturally high testosterone levels and their potential unfair advantage (Hirschberg, 2020).

## Data Availability Statement

The datasets generated during and/or analyzed during the current study are available from the corresponding author on reasonable request.

## Ethics Statement

The studies involving human participants were reviewed and approved by the local ethics committee in Stockholm (2016/1485-32, amendment 2017/779-32). The patients/participants provided their written informed consent to participate in this study.

## Author Contributions

All authors listed met the conditions required for full authorship, contributed to acquisition of the data, data analysis, interpretation of the data, and critical revision and approval of the manuscript for important intellectual content. AH, SB, and BE: concept and study design. DC: drafting of the manuscript. All authors contributed to the article and approved the submitted version.

### Conflict of Interest

AH is a medical and scientific consultant for the Swedish Olympic Committee and a member of the World Athletics, previously the International Association of Athletics Federation and the International Olympic Committee working groups on hyperandrogenic female athletes and transgender athletes.

The remaining authors declare that the research was conducted in the absence of any commercial or financial relationships that could be construed as a potential conflict of interest.
